# Supplementary material for: Mechanisms governing the pioneering and redistribution capabilities of the non-classical pioneer PU.1
Source: Nat Commun. 2020 Jan 21;11:402. doi: 10.1038/s41467-019-13960-2 (PMC6972792; doi:10.1038/s41467-019-13960-2)
Supplement: Supplementary file 7 — Source data [file 41467_2019_13960_MOESM7_ESM.zip › Source_Data/FigureS2/FigureS2B_MotifScanOutput/KmeansCluster1/homerResults/motif38.similar.html]

motif38

## Information for motif38

A
G
T
C
G
C
T
A
A
G
C
T
C
G
A
T
C
G
A
T
C
T
A
G
T
G
A
C
  
Reverse Opposite:  

A
C
T
G
A
G
T
C
G
C
T
A
C
G
T
A
T
C
G
A
C
G
A
T
T
C
A
G
  

|  |  |
| --- | --- |
| p-value: | 1e-19 |
| log p-value: | -4.396e+01 |
| Information Content per bp: | 1.782 |
| Number of Target Sequences with motif | 1958.0 |
| Percentage of Target Sequences with motif | 38.46% |
| Number of Background Sequences with motif | 12717.0 |
| Percentage of Background Sequences with motif | 32.06% |
| Average Position of motif in Targets | 100.1 +/- 57.5bp |
| Average Position of motif in Background | 99.9 +/- 68.5bp |
| Strand Bias (log2 ratio + to - strand density) | 0.1 |
| Multiplicity (# of sites on avg that occur together) | 1.27 |
| Motif File: | file (matrix) reverse opposite |

### Similar de novo motifs found

|  |  |  |  |  |  |  |  |
| --- | --- | --- | --- | --- | --- | --- | --- |
| Rank | Match Score | Redundant Motif | P-value | log P-value | % of Targets | % of Background | Motif file |
| 1 | 0.936 | A G T C G T C A A C G T A C G T C G A T C T A G T A G C C G A T | 1e-14 | -33.271086 | 11.37% | 8.02% | motif file (matrix) |
| 2 | 0.798 | C G A T G A C T C G T A A C T G A G T C G T C A C G T A T C G A G A C T | 1e-13 | -31.518329 | 9.41% | 6.44% | motif file (matrix) |
